# Motorcycle Road Traffic Injuries in a Tertiary Hospital in Nigeria: A Reflection of the Trauma Care Crisis

**DOI:** 10.7759/cureus.51141

**Published:** 2023-12-26

**Authors:** Constantine Ezeme, Emmanuel O Oladeji, Lateef A Baiyewu, Michael O Okunola, Samuel O Ogunlade

**Affiliations:** 1 Department of Surgery, University College Hospital, Ibadan, NGA; 2 Department of Surgery, Sheffield Teaching Hospitals NHS Foundation Trust, Sheffield, GBR; 3 Department of Trauma and Orthopaedics, St. Richard’s Hospital, Chichester, GBR; 4 Department of Trauma and Orthopaedics, University College Hospital, Ibadan, NGA; 5 Department of Surgery, College of Medicine, University of Ibadan, Ibadan, NGA

**Keywords:** motorcycle, two-wheeler, road traffic injury, road traffic crash, trauma, accident, nigeria, africa, low and middle country (lmic), sdg

## Abstract

Background: Motorcycle is a popular and growing form of intracity transportation in many Nigerian cities owing mainly to poorly developed transport systems. It contributes significantly to road traffic injuries (RTIs), which are a leading cause of death and disabilities in low- and middle-income countries. There is a lack of information on the quality of care received and the treatment outcome in patients with motorcycle RTIs in Ibadan and many cities in Nigeria. This study evaluated the characteristics of motorcycle-related RTIs, the quality of care received, and the outcome of the patients managed in a trauma reference center in Ibadan, Nigeria.

Methods: This is a prospective cohort study. All patients involved in motorcycle road traffic crashes who presented to the emergency department of the University College Hospital, Ibadan, between August 2020 and May 2021, were included in the study. Data on patients’ demographics, history of the crash, injuries sustained, definitive care, and the outcome of in-hospital care were obtained from patients (and/or their carers) and the medical records.

Results: A total of 156 patients were seen, out of which 74.4% were males. About 76.2% were less than 45 years with a mean age of 35.7 ± 16.3 years, and the peak age group was 18-44 years. About 37 (23.7%) patients were involved in motorcycle/motorcycle collisions, whereas 67 (42.9%) were involved in motorcycle/car collisions. Riders accounted for 59.6% (93), and although 62% (97) of the patients presented within six hours of the crash, only 10.9% (17) presented within one hour. About 48% received some form of prehospital care rendered by officials of the Federal Road Safety Corps, police officers, or passers-by, and none was attended by a dedicated emergency ambulance team. The head and the limbs were the most affected anatomical areas, while orthopedic and neurosurgical procedures were the most required emergency surgical interventions. About 66.7% were discharged home with only 21.2% of them fit to return to pre-trauma function at discharge, and the mortality rate was 17.3%. Patients who presented at 7-24 hours (AOR = 2.99; 95% CI = 1.04-8.62; p-value = 0.043) and >24 hours after the accident (AOR = 5.65; 95% CI = 1.64-19.53; p-value = 0.006) were 2.99 and 5.65 times, respectively, more likely to die from motorcycle-related accident compared to those who presented within the first six hours.

Conclusion: This study identified the growing burden of disabilities and mortalities related to motorcycle RTIs. It highlights the lack of prehospital trauma care, which is a reflection of the deficiency of a national, regional, or jurisdictional trauma system and the critical need to develop a functional trauma system.

## Introduction

Motorcycles emerged as a form of commercial intracity transportation system in Nigeria in the 1970s [1] and have remained a significant means of transportation in several cities. This has been attributed to rapid urbanization and inadequate means of transportation, a high rate of unemployment and economic difficulty, cheaper fares, the flexibility of movement, and the usage of motorcycles in areas where commercial vehicles are not accessible [1-3].

The pervasive use of commercial motorcycles, eponymously called okada in Southern Nigeria, for commuting across Nigerian cities has negative consequences. This includes an increase in criminal activities (armed robbery, kidnapping, and gang-mayhem), traffic congestion, increased environmental pollution, and road traffic crashes. These have led to the ban on commercial motorcycles in some cities across Nigeria. Despite the ban in several cities, there is a substantial increase in the motorcycle business investment in Nigeria with start-up companies like Gokada, Max.ng, SafeBoda, and Oride leading the pace [4].

An inherent disadvantage of motorcycles is their proneness to road traffic crashes (RTCs), with reports of 20 to 40 times higher fatality rates for motorcyclists when compared to car occupants [5]. A 2009 population-based survey showed that RTC associated with motorcycles accounted for about 54% of all road traffic injuries in Nigeria; the overall road traffic injury (RTI) rate was 41 per 1000 population, and mortality from RTIs was 1.6 per 1000 population [6]. Similarly, a multicenter study of almost 15,000 RTC victims in Guinea implicated motorcycles in 58.3% of their cohort [7].

Studies on the cause and consequences of motorcycle-related RTC in various regions of the country have identified poor compliance with traffic regulations [8], risky behaviors among motorcyclists, chaotic traffic, bad roads [9], lack of road safety education, and poor enforcement of traffic rules among others [10] as the causes of the increasing motorcycle-related RTCs. Interventions such as proper registration and licensing, road safety education, testing for alcohol use among motorcyclists, proper enforcement of traffic rules and regulations, the use of crash helmets, and road maintenance are being advocated as strategies for reducing motorcycle-related RTIs.

Ibadan is one of the cities in Nigeria where commercial motorcycles are still being used despite an earlier ban [11]. Like many other cities in Nigeria, Ibadan grew organically with no recourse to a formal city plan [12]. At present, no population- or hospital-based study is evaluating the pattern of injuries associated with motorcycle-related RTC and the outcome of care of victims in Ibadan and various cities in Nigeria. This lack of information tends to obscure the severity of the problem and its socioeconomic implications, limiting the attention paid to addressing this problem.

This article reports the characteristics of motorcycle-related RTIs, the quality of care received, and the outcome of the patients managed, in terms of morbidities and mortalities, in a trauma reference center in Ibadan, Nigeria. It is hoped that the data from this study will provide further insight into strategies to reduce the burden of motorcycle RTIs and enhance improved care and outcomes for victims of motorcycle-related crashes. This is a crucial step toward achieving the United Nations’ sustainable development goal (Goal 3, Target 3.6) of halving the number of deaths and injuries from road traffic accidents by 2030 [13].

## Materials and methods

This is a prospective cohort study conducted at the University College Hospital Ibadan, Nigeria, from August 2020 to May 2021. Ibadan, the capital city of Oyo state, is in South-West Nigeria and has an estimated population of 3,464,000 (2019) and is projected to be over four million in 2025 [14].

The study population included all consecutive consenting patients comprising motorcycle riders, pedestrians, and pilon riders who presented with RTI from motorcycle road traffic crashes at the Emergency Department of the University College Hospital Ibadan. All RTIs not associated with motorcycles were excluded. Relevant data was collected by one of the medical doctors in the research team using a pre-tested and validated observer-administered semistructured questionnaire. The questionnaire was developed in English and translated into local languages.

We collected data on patients’ characteristics, nature of the crash, time from crash to presentation, injuries sustained, and severity of RTI using the Injury Severity Score (ISS). Patients were then followed up throughout the course of their hospital stay to obtain details of treatments received and the outcome of in-hospital care. Ethical approval was obtained from the University of Ibadan/University College Hospital Research Ethics Review Committee (Reference No: UI/EC/20/0045).

Descriptive statistical analysis was done, while bivariate analysis and multivariate logistic regressions were performed to predict the risk of mortality from motorcycle-related RTIs with covariates, which included age, sex, level of education, type of road user, crash mechanism, speeding at the time of crash, time to presentation, length of hospital stay, severity of RTI, and type of injury. Statistical significance was set at a p-value of <0.05, and the results were presented in tables and charts. Statistical Package for the Social Sciences (SPSS) version 23 (IBM Corp., Armonk, NY) was used to analyze all data in this study.

## Results

A total of 476 patients with road traffic injuries presented to the hospital over the study period, of which 156 (32.8%) patients had motorcycle-related RTCs. The mean age of the patients was 35.7 ± 16.3, and 119 (76.2%) patients were aged < 45 years. The male-to-female ratio was 2.9:1, and 34 (21.8%) patients were commercial motorcyclists (Table 1).

**Table 1:** Patients' characteristics (N = 156) SD: Standard deviation.

Variables	Frequency (%)
Age (years)	
<18	13 (8.3)
18-44	106 (67.9)
45-64	27 (17.3)
≥65	10 (6.4)
Mean ± SD	35.7 ± 16.3
Sex	
Male	116 (74.4)
Female	40 (25.6)
Level of education	
Primary	22 (14.1)
Secondary	63 (40.4)
Post-secondary	71 (45.5)
Occupation	
Commercial motorcyclist	34 (21.8)
Others	122 (78.2)
Crash victims	
Pedestrians	21 (13.5)
Passengers	42 (26.9)
Riders	93 (59.6)

The majority of the patients were involved in a motorcycle-car crash (67; 42.9%) and motorcycle-motorcycle crash (37; 23.7%), and 61 (39.1%) reported that they were speeding at the time of the crash. About 75 (48.1%) received pre-hospital care rendered by officials of the Federal Road Safety Corps, police officers, or passers-by, and none was attended by a dedicated emergency ambulance team. Although 62% (97) of the patients presented within six hours of the crash, only 10.9% (17) were attended within one hour (Table 2).

**Table 2:** Nature of the crashes and prehospital events

Variables	Frequency (%)
Vehicles involved	
Motorcycle-car	67 (42.9)
Motorcycle-motorcycle	37 (23.7)
Lone motorcycle	20 (12.8)
Motorcycle-pedestrian	21 (13.5)
Motorcycle-large vehicle	7 (4.5)
Motorcycle-tricycle	4 (2.6)
Motorcycle speed	
High	61 (39.1)
Moderate	51 (32.7)
Low	44 (28.2)
Time from accident to presentation (hours)	
≤ 6	97 (62.2)
7-24	26 (16.7)
>24	12 (7.7)
Missing	21 (13.4)
Prehospital care	
Yes	75 (48.1)
No	81 (51.9)
Use of helmet by riders (n = 93)	
Yes	7 (7.5)
No	86 (92.5)
Number of persons on the pillion (n = 135)	
0 (lone rider)	61 (45.2)
1	45 (33.3)
2	28 (20.7)
3	1 (0.8)

The motorcycle riders were grouped as full-time commercial motorcyclists (34; 36.6%), which refers to motorcyclists whose main job is the use of motorcycles for intracity transportation for a fee, part-time commercial motorcyclists who use their motorcycle for commercial intracity transportation sometimes but have other jobs (30; 32.3%), and those who use their motorcycle for noncommercial (private) intracity movements (29; 31.1%). The median (IQR) riding experience was seven (2-10) years. About 17 (18.2%) patients reported having had formal training with a license, and seven (7.5%) wore helmets at the time of the crash.

**Figure 1:**
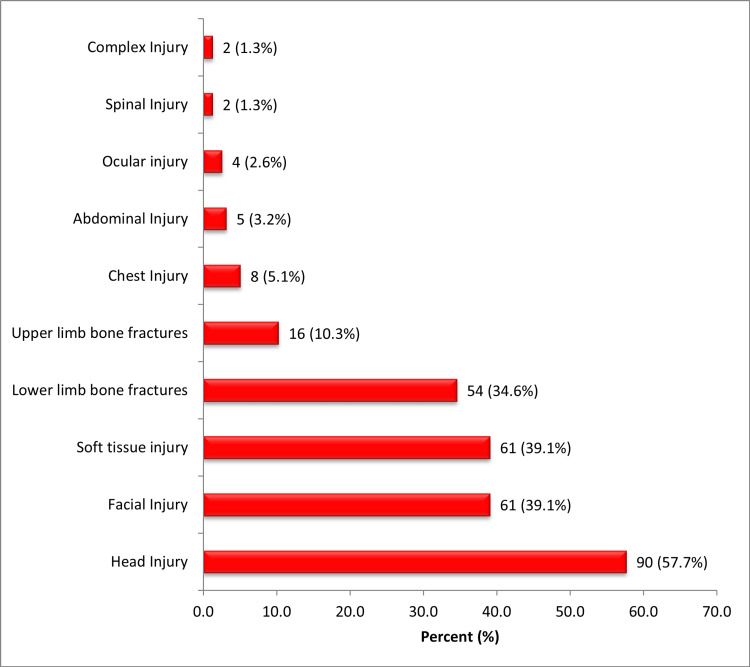
Types of injuries sustained

**Figure 2:**
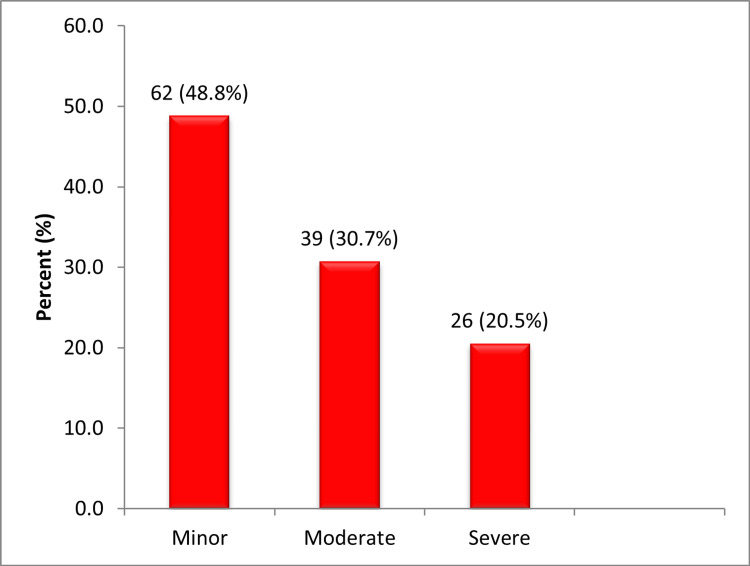
Severity of injury based on abbreviated injury scale

Head injury (90; 57.7%) and limb fractures (70; 44.9%) were the most common injuries in this cohort as shown in Figure 1. The severity of the injuries was stratified using the injury severity scale based on the abbreviated injury scale 2008 revision [15]. Twenty percent of the patients suffered severe injury (Figure 2).

Forty-two patients (26.9%) had surgical intervention; the frequency of the surgery done is shown in Table 3. The overall in-hospital outcome is depicted in Table 4. A total of 104 (66.7%) patients were discharged home, of these 82 (78.8%) were discharged with temporary disability, while 27 (17.3%) deaths were recorded (Table 4). Unadjusted data showed that speeding at the time of the crash (COR = 3.56; 95% CI = 1.21-10.46; p-value = 0.021), involvement in a motorcycle-motorcycle crash (COR = 2.72; 95% CI = 1.13-6.57; p-value = 0.026), time to presentation within 7-24 hours (COR = 2.97; 95% CI = 1.09-8.13; p-value = 0.034), and time to presentation > 24 hours (COR = 6.35; 95% CI = 2.00-20.17; p-value = 0.002) were significantly associated with mortality from motorcycle-related road traffic crash.

**Table 3:** Surgical interventions

Variables	Frequency (%)
Open reduction and internal fixation	16 (38.1)
Wound debridement	13 (31.0)
External fixation	10 (23.8)
Craniotomy and evacuation of hematoma	5 (11.9)
Exploratory laparotomy	3 (7.1)
Open reduction with K-wire fixation	2 (4.8)
Elevation of depressed skull fracture	2 (4.8)
Gardener wells traction	1 (0.6)
Elective tracheostomy	1 (2.4)
Primary wound closure	1 (2.4)
Split-thickness skin graft	1 (2.4)
Suprapubic cystostomy	1 (2.4)
Tendon repair	1 (2.4)
Wound exploration	1 (2.4)

**Table 4:** In-hospital outcomes

Variables	Frequency (%)
Overall outcome	
Discharged home	104 (66.7)
Discharged against medical advice	18 (11.5)
Referred	7 (4.5)
Died	27 (17.3)
Condition at discharged	
Fully recovered and fit to return to pretrauma function	22 (21.2)
Temporary disability may return to pretrauma function with further therapy	82 (78.8)
Length of stay (days)	
<1	45 (28.8)
1-7	36 (23.1)
8-14	26 (16.7)
>14	43 (27.6)

Adjusted data, however, showed that only time to presentation independently predicted mortality from motorcycle-related road traffic crashes. Patients who presented at 7-24 hours (AOR = 2.99; 95% CI = 1.04-8.62; p-value = 0.043) and >24 hours after the accident (AOR = 5.65; 95% CI = 1.64-19.53; p-value = 0.006) were 2.99 and 5.65 times, respectively, more likely to die from motorcycle-related accident compared to those who presented within the first six hours (Table 5).

**Table 5:** Multivariate analysis of the predictors of motorcycle-related road traffic mortality Ref = Reference. *Significant values.

Variables	AOR	95% CI		p-value
		Lower	Upper	
Speeding at the time of the crash				
No	Ref			
Yes	2.98	1.09	10.21	0.057
Motorcycle-motorcycle clash				
No	Ref			
Yes	2.59	0.97	6.93	0.058
Time from accident to presentation (hours)				
≤6	Ref			
7-24	2.99	1.04	8.62	0.043*
>24	5.65	1.64	19.53	0.006*

## Discussion

This study described the characteristics of motorcycle-related RTIs, the scope of treatment including the apparent lack of prehospital trauma care, and the outcome profile among crash victims managed at a tertiary hospital in a lower-middle-income country (LMIC). It identifies the growing burden of motorcycle RTI, deaths, and disability, in parallel to the rapidly rising preference for motorcycles as a major means of intracity goods and passenger transportation or commuting.

Motorcycle-car collisions were the most common cause of RTI in this study with most patients being riders and within the young, economically vibrant age group of 18-44 years. Previous studies have reported similar sociodemographic characteristics and collision mechanisms among motorcycle-related crash victims [9,16,17].

The median riding experience was seven years, which was higher than the three years reported by Oluwadiya et al. and Olasinde et al. [9,18]. Nevertheless, the injury severity and mortality rate from our study were significantly higher. Only 17 (18.2%) patients had received formal training with a license, while seven (7.5%) wore helmets at the time of the crash, which is comparable to previously reported findings and reflects the wanton lack of regulation of motorcyclists and the prevalent disregard for road safety guidelines [9,16].

Head injury was the most common injury from our study cohort, which is at variance with most studies that reported long bone fractures as the most prevalent injury [9,16,17], but is in keeping with a recent study conducted in urban Kenya [19]. This pattern of injury burden may be a consequence of the preponderant motorcycle-car collision mechanism of injury and their association with traumatic brain injury from head-leading impacts [20].

Sisimwo et al. reported mild to moderate injuries in the vast majority of their study population, comparable to findings from our study; however, fewer patients sustained severe injuries in their cohort - 16.1% as against 20.5% [21]. Surgical intervention was indicated in 26.9% of patients, predominantly for fracture fixation, which was significantly lower than the reports of up to 87.5% from previous studies [19,21,22]. However, these studies had a broader scope of surgical procedures, including wound dressing and suturing, which are often performed in the emergency department in our hospital and therefore not categorized as surgical intervention requiring theater.

Only 21.2% of the patients in this study were fit to return to pre-trauma function at discharge. Residual functional impairment, extended periods of rehabilitation, and reduced health-related quality of life constitute significant socioeconomic consequences for patients, families or carers, and society [23]. The effects are even more devastating in Nigeria and other low- and middle-income countries where most patients make out-of-pocket payments for trauma care, with little financial protection mechanisms available both for healthcare financing and injury compensation claims [24,25].

The mortality rate of 17.3% is a striking finding from our study when compared to previous reports of 6.3%, 7.1%, and 9% by Dongo et al., Solagberu et al., and Saidi et al., respectively [16,22,26], but resonates with the rate of 16.7% in Tanzania and 16.1% according to the Nigerian Federal Road Safety Corps [27,28]. The high mortality rate reported in this study mirrors the preponderance of head injury, which is the leading cause of death from motorcycle-related RTCs, even in helmeted individuals [29].

Although overspeeding at the time of the crash, involvement in a motorcycle-motorcycle crash, and time to presentation above six hours were significantly associated with mortality from motorcycle-related road traffic crashes, time of presentation above six hours after the crash was the only independent predictor. A majority of the patients presented within six hours but after the one-hour mark and received no pre-hospital care, which reinforces the lack of organized and functional prehospital emergency services in Nigeria [30,31]. This worrying trend was highlighted by Ibrahim et al. in a retrospective review of 23,537 patients managed at a tertiary hospital in an urban area, which found that only 2.3% received formal prehospital care [30]. This gross deficiency of prehospital care is a partial reflection of the nonexistent trauma system on a national scale and the tragic failure of the emergency medical services established by the jurisdictional state governments [32].

Other authors have alluded to the association of late presentation with worse clinical outcomes while reporting additional predictors of mortality, which include older age of the patient, non-use of helmet, hypotension on admission, severe head injury, injury severity (major trauma), low Glasgow Coma Scale score, and need for intensive care unit admission [19,22,28].

This study clearly identifies the need to improve prehospital trauma care through the development of a functional national trauma system, which has proven to ensure efficient utilization of resources and enhanced outcomes for victims of RTCs in high-income countries. Recommendations proposed by Oyedokun et al. toward improving infrastructure, communication, integration of emergency services, and instituting relevant policies backed by adequate funding offer a blueprint for addressing this chronic problem [32]. Additionally, there is a need to implement strategies to make the roads safer amidst the rapidly increasing number of motorcycles on Nigerian roads through proper enforcement of appropriate motorcycle rider qualification and licensing processes, compliance with traffic rules and regulations, improvement of road infrastructure, and development of safer urban transport systems.

Limitations

This study was conducted in a major trauma reference hospital where most of the severely injured patients from road traffic accidents within the Ibadan metropolis would typically be referred; however, patients with minor injuries may seek care at lower-level healthcare facilities. Additionally, the absence of prehospital ambulance services implies that prehospital death records remain largely unknown. Hence, the obtainable data may have underestimated motorcycle RTIs. Nevertheless, this prospective study highlights an important public health problem, and the challenges described are shared by other low- and middle-income countries.

## Conclusions

This study identified the growing burden of motorcycle RTIs, deaths, and life-wrecking disabilities. It highlights the need for a national strategy to develop a functional trauma system that engenders efficient emergency ambulance services and a prehospital care system for road crash victims. Additionally, there is an imperative to develop organized urban transport systems to meet up with the unrelenting pace of urbanization experienced on a national scale. Significant head injury reported from this study also calls for rekindled efforts to revisit the enforcement of helmet laws for riders and passengers, in addition to the implementation of appropriate motorcycle rider qualification and licensing processes.
